# Front-Line vs Second-Line Healthcare Workers: Susceptibility Prediction to COVID-19 Infection in a Tertiary Care Teaching Institute

**DOI:** 10.7759/cureus.37915

**Published:** 2023-04-21

**Authors:** Manuj K Sarkar, Thirunavukkarasu Arun Babu, Subhra Dey, Rakesh Upparakadiyala, Purushotham Lingaiah, Vinayagamoorthy Venugopal

**Affiliations:** 1 General Medicine, All India Institute of Medical Sciences, Deoghar, Deoghar, IND; 2 Pediatrics, All India Institute of Medical Sciences, Mangalagiri, Mangalagiri, IND; 3 Dentistry, All India Institute of Medical Sciences, Deoghar, Deoghar, IND; 4 General Medicine, All India Institute of Medical Sciences, Mangalagiri, Mangalagiri, IND; 5 Orthopaedics, All India Institute of Medical Sciences, Mangalagiri, Mangalagiri, IND; 6 Community and Family Medicine, All India Institute of Medical Sciences, Deoghar, Deoghar, IND

**Keywords:** sars-cov-2 (severe acute respiratory syndrome coronavirus-2), front-line healthcare worker, second-line healthcare worker, covid susceptibility, covid appropriate behaviour, covid-19

## Abstract

Background

Since the beginning of the novel coronavirus disease in Wuhan city of China in 2019 and its spreading worldwide and taking the form of a pandemic, many healthcare workers (HCWs) were affected by coronavirus disease 2019 (COVID-19) infection. Though we have used many types of personal protective equipment (PPE) kits while taking care of COVID-19 patients, we have seen COVID-19 susceptibility in different working areas were different. The pattern of infection in different working areas depended on HCWs following COVID-19 appropriate behavior. Therefore, we planned to estimate the susceptibility of front-line HCWs and second-line HCWs to getting COVID-19 infection.

Aim

To determine the risk of COVID-19 in front-line healthcare workers as compared to second-line healthcare workers.

Method and materials

We planned a retrospective cross-sectional analysis of COVID-19-positive healthcare workers from our institute within six months. Their nature of duty was analyzed and they were divided into two groups: 1) Front-line HCWs were defined as those who were working or who have worked in screening areas of the outpatient department (OPD) or COVID-19 isolation wards within the prior 14 days and provided direct care to patients with confirmed or suspected COVID-19. 2) Second-line HCWs were those who were working in the general OPD or non-COVID-19 areas of our hospital and did not have contact with COVID-19-positive patients.

Results

A total of 59 HCWs became COVID-19 positive during the study period, 23 as front-line and 36 as second-line HCWs. The mean (SD) duration of work as a front-line worker was 51 and as a second-line worker was 84.4 hours. Fever, cough, body ache, loss of taste, loose stools, palpitation, throat pain, vertigo, vomiting, lung disease, generalized weakness, breathing difficulty, loss of smell, headache, and running nose were present in 21 (35.6%), 15 (25.4%), 9 (15.3%), 10 (16.9%), 3 (5.1%), 5 (8.5%), 5 (8.5%), 1 (1.7%), 4 (6.8%), 2 (3.4%), 11 (18.6%), 4 (6.8%), 9 (15.3%), 6 (10.2%) and 3 (5.1%), respectively. To predict the risk of getting COVID-19 infection in HCWs, binary logistic regression with COVID-19 diagnosis as the output variable was modeled with hours of working in COVID-19 wards as front-line and second-line workers as independent variables. The results showed that there was a 1.18 times increased risk of acquiring the disease for every one-hour excess of working as a front-line worker, whereas, for second-line workers, it was slightly lower, with a 1.11 times increased risk for developing COVID-19 disease with every one hour increase in duty hours. Both these associations were statistically significant (p=0.001 for front-line and 0.006 for second-line HCWs).

Conclusion

COVID-19 has taught us the importance of COVID-19 appropriate behavior in preventing the spread of respiratory organisms. Our study has shown that both the front-line and second-line HCWs are at increased risk of getting the infection and proper use of a PPE kit or mask can decrease the spread of such respiratory pathogens.

## Introduction

The novel coronavirus disease 2019 (COVID-19) caused by severe acute respiratory syndrome coronavirus 2 (SARS-CoV-2) started in Wuhan City, China. Soon World Health Organization (WHO) declared it a pandemic on March 11, 2020. Several mechanisms of rapid spread were proposed, soon widespread locked down, quarantine, isolation of cases, contact tracing, and extensive testing for early detection, and every attempt was made to contain the spread of the virus [1].

Spread of the virus can be from cases to contacts, asymptomatic or pre-symptomatic cases to contacts, in the hospital setting from cases to healthcare workers (HCWs), HCWs to HCWs in the hospital, HCWs to family members of HCWs or even community transmission [2-5]. Asymptomatic carriers can transmit the virus in up to 40-45% of cases [3-5]. The prevalence of infection in HCWs was 11%, most frequently in nurses (48%) and physicians (25%). Infection is more common in inpatient departments and non-emergency wards rather than intensive care facilities, HCWs in non-COVID-19 areas are more susceptible to infection because of their reluctance in following COVID-19-appropriate behavior [2]. A similar finding of a 0.5% infection rate in front-line HCWs and 1.4% in second-line HCWs was reported by Lai X et al. They also found the prevalence of subclinical infection in asymptomatic front-line HCWs to be 0.74% and 1% in second-line HCWs [6]. During the initial part of the pandemic, there was no difference in infection of HCWs in various exposure areas, but later, infection rates in HCWs in hospitalization/non-emergency areas were found more compared with other areas suggestive of more contact time for the transmission of the virus or differences in following a COVID-19-appropriate protocol, which can be defined as maintaining social distance, wearing a properly fitted mask, avoiding unrequired contact, wearing personal protective equipment (PPE) kit, etc. for high transmission zones as per guidelines [7-10]. Because of the excessive use of PPE kits and the fear of COVID-19 infection, HCWs also face anxiety, depression, post-traumatic stress disorder, and mental stress [11]. Recurrent infection also increased their stress level. Atypical symptoms also played a very important role in causing stress to HCWs, which affects COVID-19-appropriate behavior [12,13]. Regular use of PPE is important to reduce nosocomial transmission to HCWs [14], as COVID-19 can have a lot of asymptomatic carriers, who can transmit easily and may lead to the super spread of infection in hospitals [15]. Front-line HCWs are at high risk of infection (10-20% of all cases of COVID-19 infections) because of their proximity to COVID-19-positive patients [16-18]. Thus, all these studies have found the changing nature of the risk of COVID-19 infection to front-line vs second-line HCWs. Therefore, we planned to compare the susceptibility of front-line HCWs and second-line HCWs to COVID-19 infection so that appropriate measures or guidelines can be provided to HCWs regarding the use of appropriate PPE.

## Materials and methods

Study setting and design

We planned a retrospective study of the duty schedule of COVID-19-positive HCWs while working in AIIMS, Mangalagiri, during the study period of six months, from July 1, 2020, to December 31, 2020. This study was approved by the Institutional Ethical Committee of AIIMS, Mangalagiri, with the approval number AIIMS/MG/IEC/2020-21/75.

Study participant

We included all eligible HCWs working in AIIMS, Mangalagiri, including doctors, nurses, housekeeping staff, male and female nursing orderlies, security guards, etc., available during the study period. Persons who denied giving consent were excluded. Front-line HCWs were defined as those who were working or who have worked in screening areas of the outpatient department (OPD) or COVID-19 isolation wards within the prior 14 days and provided direct care to patients with confirmed or suspected COVID-19. Second-line HCWs were those who were working in the OPD or non-COVID-19 areas of our hospital and who did not have contact with COVID-19-positive patients.

Sample size and data collection

We took written informed consent for participating in the study prior to enrolling them. We collected data based on their duty schedule and the type of duty they performed during the study period, and we divided them into two groups: 1) front-line HCWs and 2) second-line HCWs. Their duty shifts, i.e., the number of hours, for each group was calculated. All the HCWs who became COVID-19 positive during the study period, their average exposure, and their demographic information were collected by a structured questionnaire, and the clinical symptomatology, laboratory, and radiologic information were collected from electronic medical records and other available reports. We gave treatment based on a clinical category of disease such as mild, moderate, and severe disease. We securely stored all collected data as soft copies in the computer.

Statistical analysis

We entered the data in Microsoft Office Excel (Microsoft Corporation, Redmond, WA) and analyzed using SPSS statistical software for Windows, Version 21.0. (Armonk, NY: IBM Corp). Continuous variables were described using means (standard deviation). We described categorical variables as frequency and percentages. The chi-square test was used to find out the association between demographic and clinical parameters with COVID-19 infection status. We modeled multivariate binary logistic regression to identify the risk for front-line and second-line workers to develop COVID-19 infection. The strength of association was presented with a 95% confidence interval. A two-sided p-value of less than 0.05 was considered statistically significant.

## Results

There were 59 HCWs who became COVID-19 positive during the study period, of them, 23 (38.9%) were front-line workers and 36 (61.1%) were second-line workers. All 59 HCWs had done duty as second-line workers and only 23 had worked as front-line workers in COVID-19 wards. The mean (SD) duration of work as a front-line and second-line worker was 51 (23.1) and 84.4 (27) hours, respectively. The average (SD) age of the study participants was 31.9 (6.5) years. Of the total 59 study participants, 42 (71.2%) were males. Overall, 25 (42.4%) had symptoms suggestive of COVID-19 following exposure. Fever was present in 21 (35.6%) of the participants. Out of them, 10 (47.6%) HCWs were front-line workers. The risk of COVID-19 infection in front-line HCWs was higher for those who had a fever (47.6%) than those who did not present with a fever (34.2%); however, this association was statistically non-significant. Cough, body ache, loss of taste, loose stools, palpitation, and throat pain were present in 15 (25.4%), 9 (15.3%), 10 (16.9%), 3 (5.1%), 5 (8.5%), and 5 (8.5%) HCWs, respectively. Participants with all these symptoms had a higher risk of COVID-19 infection than front-line HCWs. Of these symptoms, loose stools and throat pain had a statistically significant association for front-line HCWs (p=0.03 & p=0.04, respectively). Vertigo, vomiting, and lung disease were seen in 1 (1.7%), 4 (6.8%), and 2 (3.4%) participants, respectively. They also had a higher risk but a statistically non-significant association for front-line HCWs. Clinical symptoms like generalized weakness, breathing difficulty, loss of smell, headache, and running nose were present in 11 (18.6%), 4 (6.8%), 9 (15.3%), 6 (10.2%), and 3 (5.1%) numbers of HCWs with statistically non-significant higher risk for second-line HCWs. We gave treatment based on the clinical category of disease (mild disease {A=56 (94.9%)}, Moderate disease {B=3 (5.1%)}) (Table 1).

**Table 1:** Demographic and clinical characteristics of study participants diagnosed with COVID-19 (N=59) Note: ! column percentage, ^ row percentage, # p value based on the chi-square test, * statistically significant (p<0.05)

Sl. No.	Characteristics	Total participants N=59 (%^!^)	Covid diagnosis Mean (SD) / n (% ^)		p value#
			Front-line HCW (n=23)	Second-line HCW (n=36)	
1	Mean Age in years (SD)	31.9 (6.5)	32.5 (5.8)	31.0 (7.5)	0.40
2	Gender				
	Male	42 (71.2)	18 (42.9)	24 (57.1)	0.34
	Female	17 (28.8)	5 (29.4)	12 (70.6)	
3	Presence of Symptom				
	Symptomatic	25 (42.4)	10 (40)	15 (60)	0.89
	Asymptomatic	34 (57.6)	13 (38.2)	21 (61.8)	
4	Fever				
	Absent	38 (64.4)	13 (34.2)	25 (65.8)	0.19
	Present	21 (35.6)	10 (47.6)	11 (52.4)	
5	Cough				
	Absent	44 (74.6)	15 (34.1)	29 (65.9)	0.18
	Present	15 (25.4)	8 (53.3)	7 (46.7)	
6	Generalized weakness				
	Absent	48 (81.4)	19 (39.6)	29 (60.4)	0.84
	Present	11 (18.6)	4 (36.4)	7 (63.6)	
7	Body ache				
	Absent	50 (84.7)	17 (34)	33 (66)	0.06
	Present	9 (15.3)	6 (66.7)	3 (33.3)	
8	Breathing Difficulty				
	Absent	55 (93.2)	22 (40)	33 (66)	0.55
	Present	4 (6.8)	1 (25)	3 (75)	
9	Loss of Smell				
	Absent	50 (84.7)	20 (40)	30 (60)	0.71
	Present	9 (15.3)	3 (33.3)	6 (66.7)	
10	Loss of Taste				
	Absent	49 (83.1)	17 (34.7)	32 (65.3)	0.13
	Present	10 (16.9)	6 (60)	4 (40)	
11	Loose Stools				
	Absent	56 (94.9)	20 (35.7)	36 (64.3)	0.03*
	Present	3 (5.1)	3 (100)	0	
12	Headache				
	Absent	53 (89.8)	21 (39.6)	32 (60.4)	0.76
	Present	6 (10.2)	2 (33.3)	4 (66.7)	
13	Palpitation				
	Absent	54 (91.5)	20 (37)	34 (63)	0.31
	Present	5 (8.5)	3 (60)	2 (40)	
14	Running nose				
	Absent	56 (94.9)	23 (41.1)	33 (58.9)	0.15
	Present	3 (5.1)	0	3 (100)	
15	Throat pain				
	Absent	54 (91.5)	19 (35.2)	35 (64.8)	0.04*
	Present	5 (8.5)	4 (80)	1 (20)	
16	Vertigo				
	Absent	58 (98.3)	22 (37.9)	36 (62.1)	0.21
	Present	1 (1.7)	1 (100)	0	
17	Vomiting				
	Absent	55 (93.2)	21 (38.2)	34 (61.8)	0.64
	Present	4 (6.8)	2 (50)	2 (50)	
18	Lung Disease				
	Absent	57 (96.6)	21 (36.8)	36 (63.2)	0.07
	Present	2 (3.4)	2 (100)	0	
19	Category of Treatment				
	A	56 (94.9)	22 (39.3)	34 (60.7)	0.83
	B	3 (5.1)	1 (33.3)	2 (66.7)	

In order to predict the risk of acquiring COVID-19 due to working as front-line health care workers as compared to second-line workers in the wards where COVID-19 patients were admitted, binary logistic regression with COVID-19 diagnosis as the output variable was modeled with hours of working in COVID-19 wards as front-line and second-line workers as independent variables. The results showed that there was a 1.18 times increased risk of acquiring the disease for every one-hour excess of working as a front-line worker. The risk for second-line workers was slightly lower than that of 1.11 times for developing COVID-19 disease. Both these associations were statistically significant (p=0.001 for first-line and 0.006 for second-line workers) (Table 2).

**Table 2:** Prediction of COVID-19 infection as per the duration of working hours using multivariate binary logistic analysis (N=59) Note: #p value based on multivariate binary logistic regression. * Statistically significant (p<0.05).

Predictors	Beta Co-efficient	Adjusted Odd’s Ratio	95% Confidence Interval		p value#
			Lower	Upper	
Duration of work as front-line workers	0.167	1.18	1.07	1.30	0.001*
Duration of work as second-line workers	0.111	1.11	1.03	1.21	0.006*

## Discussion

Our study showed that front-line HCWs are more likely to contact COVID-19 by 1.18 times with every one-hour increase in duty hours than 1.11 times for second-line HCWs. COVID-19 transmission potential is equal both in symptomatic and asymptomatic individuals because of the similar number of viral loads found in both conditions, as stated by Zou L et al. [19]. Therefore, people who are in the asymptomatic stage and visit the hospital for non-COVID-19-related causes can transmit the virus to HCWs working in second-line areas. A study conducted by JC Yombi et al. stated that there is a high incidence of subclinical infection and asymptomatic cases in COVID-19 cases, increasing the risk of COVID-19 infection in HCWs [20]. Chow et al. studied many common but non-specific symptoms that can be missed during screening for COVID-19 [21]. Screening based on fever, cough, breathlessness, and sore throat can miss 17% of positive cases. If chills and myalgia are included, still we may miss 10% of cases of COVID-19 [20,21].

Using PPE can reduce the infections of HCWs, but there is no clear-cut guideline for which type of PPE to be used in which situation. Therefore, the availability of clear-cut guidelines, proper instructions for donning and doffing, fit testing, training of HCWs, and the attitude of HCWs to follow the instructions, these factors play an important role in limiting the spread of the virus to HCWs and the community [14,22-25]. HCWs working in non-COVID-19 areas have a lower likelihood of using PPE or following the appropriate COVID-19 protocols [9,22]. Though there is no study to compare the risk and infection rate of HCWs in different zones of the hospital, our study has shown that HCWs are at increased risk of getting COVID-19 infection and the risk is more for front-line HCWs than the second-line HCWs and the difference is statistically significant. All HCWs should follow COVID-19-appropriate behavior all the time, irrespective of the type of duty they are performing.

Many published articles and guidelines have reported that there was a shortage of PPE kits to be used by HCWs as per the recommended policy during the pandemic, and it is still going to be a problem if they face a similar situation in the future. The availability of PPE kits for proper management of pandemics or similar situations is very important and people need to follow the guidelines issued by the competent authority [9,26-27]. World Health Organisation has guided and recommended different PPE kits in different areas of hospitals based on types of activity in the hospital [23,28,29].

The risk of COVID-19 is threefold more among front-line healthcare workers as compared with the general community but the evidence on COVID-19 risk to HCWs based on the type of work is lacking as reported by LH Nguyen et al. [30]. Our study showed that HCWs working in hospitals are at increased risk of getting the infection, and front-line HCWs are at increased risk compared with second-line HCWs. This finding may explain the importance of COVID-19 protocol being followed in all areas of the hospital. Many probable factors like unavailability of PPE kits for second-line workers: negligence in following COVID-19 protocol while on non-COVID-19-related duty, and less protective equipment used by second-line HCWs, can play a causative role in the high risk of infection in non-COVID-19 areas.

Limitations of the study

This study finding has shown that the susceptibility of COVID-19 to HCWs is high and the difference between front-line and second-line HCWs is found to be statistically significant, but there were 59 HCWs who suffered from COVID-19 during the study period, which restricted our sample size. A similar study with a larger sample size will provide more reliable study results. We included HCWs who suffered from COVID-19 to extract their duty schedule and nature of exposure. Other study designs like cohort studies, case-control studies, randomized control trials, etc. would have provided more reliable outcomes.

## Conclusions

The COVID-19 pandemic has taught us many things including the importance of COVID-19 appropriate behavior, the importance of using PPE kits in preventing the spread of COVID-19 and other illnesses prone to spread by airborne, droplet transmission, contact transmission, including SARS, Middle East Respiratory Syndrome Coronavirus (MERS CoV), influenza (swine flu), and other respiratory viral illness. Our study showed us that though front-line HCWs are at higher risk than second-line HCWs of getting an infection and suffering from a disease like COVID-19, second-line HCWs are also at higher risk with each hour increase in duty. Therefore, COVID-19 appropriate behavior like maintaining social distancing, use of masks, use of hand sanitizers, not touching anything when not required, etc. are vital in preventing infections in HCWs irrespective of their type of duty. Proper use of PPE kits by all HCWs as per standard guidelines is essential in preventing hospital-acquired infection in any disease, which can be transmitted by respiratory pathways.
